# Evaluation of E-Cigarette Liquid Vapor and Mainstream Cigarette Smoke after Direct Exposure of Primary Human Bronchial Epithelial Cells

**DOI:** 10.3390/ijerph120403915

**Published:** 2015-04-08

**Authors:** Stefanie Scheffler, Hauke Dieken, Olaf Krischenowski, Christine Förster, Detlev Branscheid, Michaela Aufderheide

**Affiliations:** 1 Cultex Laboratories GmbH, Feodor-Lynen-Str. 21, 30625 Hannover, Germany; E-Mails: h.dieken@cultex-laboratories.com (H.D.); o.krischenowski@cultex-laboratories.com (O.K.); m.aufderheide@cultex-laboratories.com (M.A.); 2 Institute of Pathology, KRH Klinikum Nordstadt, Haltenhoffstr. 41, 30167 Hannover, Germany; E-Mail: christine.foerster@krh.eu; 3 Department of Thoracic Surgery, Bielefeld Evangelical Hospital, Burgsteig 13, 33617 Bielefeld, Germany; E-Mail: detlev.branscheid@evkb.de

**Keywords:** electronic cigarette, smoking, tobacco, nicotine, cytotoxicity, normal bronchial epithelial cells, air-liquid interface, CULTEX^®^ RFS, public health

## Abstract

E-cigarettes are emerging products, often described as “reduced-risk” nicotine products or alternatives to combustible cigarettes. Many smokers switch to e-cigarettes to quit or significantly reduce smoking. However, no regulations for e-cigarettes are currently into force, so that the quality and safety of e-liquids is not necessarily guaranteed. We exposed primary human bronchial epithelial cells of two different donors to vapor of e-cigarette liquid with or without nicotine, vapor of the carrier substances propylene glycol and glycerol as well as to mainstream smoke of K3R4F research cigarettes. The exposure was done in a CULTEX^®^ RFS compact module, allowing the exposure of the cells at the air-liquid interface. 24 h post-exposure, cell viability and oxidative stress levels in the cells were analyzed. We found toxicological effects of e-cigarette vapor and the pure carrier substances, whereas the nicotine concentration did not have an effect on the cell viability. The viability of mainstream smoke cigarette exposed cells was 4.5–8 times lower and the oxidative stress levels 4.5–5 times higher than those of e-cigarette vapor exposed cells, depending on the donor. Our experimental setup delivered reproducible data and thus provides the opportunity for routine testing of e-cigarette liquids to ensure safety and quality for the user.

## 1. Introduction

In the early 2000s, electronic cigarettes were introduced as an aid for smoking cessation and replacement and in the last few years, the consumption has risen significantly. In 2012, about 288 different e-cigarette brands were available online, whereas this number increased to 466 in January 2014 [1]. Studies have shown, that the usage of e-cigarettes can help smokers not intending to quit to significantly reduce their cigarette consumption or even stop entirely [2,3,4].

However, the regulation and control of e-cigarette liquids is neither standardized nor adequate to ensure sufficient quality and safety for the user. Former studies have shown that the labeling and the actual content can differ significantly, for example with regard to nicotine concentrations deviating from the declaration or nicotine-presence in nicotine-free labeled liquids. Furthermore, toxic substances such as nitrosamines and diethylene glycol as well as pharmacological components like rimonabant and aminotadalafil have been found in e-cigarette liquids [5,6,7].

Electronic cigarette liquids generally consist of a mixture of glycerol and propylene glycol (both used as carrier substances), flavorings and optionally, variable concentrations of nicotine. Although all ingredients, with exception of nicotine, should be approved as food additives, their harmlessness is not proven, since their approval is not necessarily meaningful for their inhalational use. The butterscotch flavor diacetyl for example, which is safe to eat but leads to a severe lung condition known as “popcorn lung” or bronchiolitis obliterans when inhaled, has been found in e-cigarette liquids [8]. Besides that, the effects of the heated ingredients are unknown. 

Only few studies are available about the health effects of e-cigarettes, which can be divided into chemical, clinical and toxicological studies. In chemical studies, the liquids are analyzed for their ingredients, but mostly only toxicants known from cigarette smoke are investigated, and the search for other unknown, possible toxic compounds is often not included. In clinical trials, users are examined after short-term usage of e-cigarettes with regard to respiratory function and cardiovascular system responses. However, long-term studies indicating possible developments of clinically evident diseases are missing. Toxicological studies comprise a few animal experiments about the effects of inhaled glycerol or propylene glycol and some *in vitro* studies using established cell lines [9]. These studies comprise exposure of human embryonal stem cells, human pulmonary fibroblasts and murine neuronal stem cells to e-cigarettes liquids as well as exposure of fibroblasts and cardiomyoblasts to e-cigarette vapor extracts [10,11,12,13].

Although these investigations have suggested cytotoxic effects of several e-liquids, the significance of the results is limited, since the cell types used in those studies are not in direct contact with e-cigarette liquid in the human body. The primary target organ of e-cigarette vapor is the respiratory tract and therefore, lung-derived cell cultures should be the *in vitro* model of choice. 

For this purpose, in this study we exposed primary normal human bronchial epithelial (NHBE) cells directly to vapor of two different e-cigarette liquids (0% and 2.4% nicotine). The cells were cultivated on semi-permeable membranes of cell culture inserts and are kept air-lifted in the exposure device, a CULTEX^®^ RFC compact module. The cell surface is exposed to the surrounding atmosphere and the nutrient supply is realized from the basal side of the cells. We exposed the cultures to e-liquid vapor from 200 puffs and analyzed cell viability and the oxidative stress level in the cells 24 h post exposure. Furthermore, we exposed cells to vapor of the carrier substances glycerol and propylene glycol using the same puff profile and puff numbers. Clean air exposed cells and cells left air-lifted in the incubator were used as positive controls as well as cells exposed to mainstream smoke of 10 cigarettes as negative controls. 

## 2. Materials & Methods

### 2.1. E-Liquids & Cigarettes

The tested refill e-liquids were purchased from Johnsons Creek (Hartland, WI, USA). The e-liquids with the flavor “Tennessee Cured” are propylene glycol based and have nicotine concentrations of 0.0% and 2.4% (24 mg/mL). The ingredients of the liquid are listed on the bottle and are as followed:

USP grade propylene glycol, USP grade vegetable glycerol, deionized water, natural flavors, artificial flavors, USP grade nicotine (not in 0.0%), USP grade citric acid (as a preservative).

Propylene glycol and glycerol were obtained from Alfa Aesar (Karlsruhe, Germany), with a purity of 99.5%. For cigarette smoke exposure, K3R4F research cigarettes (University of Kentucky, Lexington, KY, USA) with a standard cellulose acetate filter tip were used.

### 2.2. Cell Isolation & Cultivation

Normal human bronchial epithelial (NHBE) cells were isolated from ring-shaped bronchus samples of two different donors, and the obtained cells were named NHBE48 and NHBE33. The samples were received from a male patient (age 69) with a lung adenocarcinoma in the right upper lobe (NHBE33) and from of a 75-year old cancer patient during a wedge resection of the upper lobe (NHBE48). Both samples were obtained from the Bielefeld Evangelical Hospital (Bielefeld, Germany). In accordance with the Declaration of Helsinki, both subjects gave their informed consent to the research use of the removed lung tissue samples. 

Upon arrival in our laboratory, the bronchus samples were incubated for 24 h at 4 °C on a rocking platform in incubation medium (MEM medium containing dithiothreitol (0.5 mg/mL), DNase (10 µg/mL) and antibiotics (40 µg/mL tobramycin, 50 µg/mL vancomycin, 50 µg/mL ceftazidime, 2.5 µg/mL amphotericin B, 50 U/mL penicillin/streptomycin)). Afterwards, the ring shaped samples were transferred into a PBS-containing Petri dish, opened longitudinally, isolated from residual parenchyma and cut into smaller pieces of approximately 8 × 5 mm. The bronchus pieces were then placed into cryovials, containing DMEM with 10% FCS and 10% DMSO and frozen to −80 °C. After storage at −80 °C overnight, the vials were moved to a liquid nitrogen tank and stored until needed. 

For cell isolation, the samples were thawed in a water bath at 37 °C, transferred into Petri dishes and rinsed with PBS after removal of the freezing medium. Incubation medium containing 0.1% protease XIV was added and the samples were incubated for 1–2 h at 4 °C on a rocking platform. Afterwards, bronchial epithelial cells were isolated by thoroughly scraping the luminal surface of the bronchus pieces with a scalpel. 

The cell suspensions were homogenized, pipetted into centrifugation tubes and centrifuged for 10 min at 170× *g*. The resulting cell pellets were resuspended in 4.5 mL–9 mL AEGM medium. The cell suspensions of each sample were then equally divided to two collagen IV coated wells of a 6-well plate to grow in culture. 

After the first passage, NHBE cells were cultivated in collagen IV coated culture flasks using AEGM Medium. After reaching 80%–90% confluence, the cells were seeded on collagen IV coated cell culture inserts (seeding density: 2.1 × 10^5^/cm^2^). The cells were cultivated under submerged conditions and supplied with AEGM medium for 1 day before the apical medium was removed and the cells were transferred to the exposure module. The exposure experiments were performed with cells of passages 2–4. This procedure was identical for cells of both donors. 

MEM medium was obtained from Lonza (Basel, Switzerland); PBS, penicillin/streptomycin, DMEM from Biochrom, (Cambridge, UK) and AEGM medium from Promocell (Heidelberg, Germany). All other cell culture reagents were purchased from Sigma Aldrich (St. Louis, MO, USA). 

### 2.3. Exposure

All exposure experiments were performed in a CULTEX^®^ RFS compact module (Cultex Laboratories GmbH, Hannover, Germany), exposing six cell culture inserts at a time in each experimental run. For e-cigarette vapor experiments, a Reevo Mini-S (In-Smoke, Winnenden, Germany) was used, equipped with a 3.3 V/900 mAh battery and a vaporizer with a resistance of 2.2 Ohm. The e-cigarette was connected to the piston pump of a smoking robot and 200 puffs were taken with a puff volume of 35 mL, a puff duration of 2 s and a blow-out time of 7 s. The smoking robot was operated in asynchronous mode, meaning that puffs were taken successively, so that the cells were surrounded by vapor during the whole exposure time. For a better distribution, the e-liquid vapor was diluted with synthetic air (1 L/min) before sucked into the CULTEX^®^ RFS compact via a vacuum pump with a flow rate of 5 mL/min/insert. The exposures to pure glycerol and propylene glycol were performed using the same puff parameters and puff numbers. 

For mainstream smoke exposure, 10 K3R4F cigarettes were smoked by the smoking robot using the same parameters as described for the e-cigarette. Each cigarette was puffed six times. The freshly generated main stream smoke was equally diluted with synthetic air (1 L/min) and also entered the CULTEX^®^ RFS compact with a rate of 5 mL/min/insert. The clean air exposure (clean air control) was performed for 30 min with the flow rates described above. 

The flow rates were controlled by mass flow controllers (IQ+ Flow and EL-Flow Select, Bronckhorst, Ruurlo, The Netherlands). The exhaust air was directed back to the fume hood. 

As a second control, cell cultures were used that remained air-lifted in the incubator for the exposure time (incubator control). 

### 2.4. Analysis

The analyses were done 24 h after the exposure to allow the cells to respond to the exposure. In order to analyze cell viability and oxidative stress in the same cell, two cell-based assays were combined. The oxidative stress was analyzed first, using the ROS-Glo™ H_2_O_2_ Assay (Promega, Madison, WI, USA). Afterwards, cell viability was measured with the CellTiter-Blue^®^ Assay (Promega).

For the ROS-Glo™ H_2_O_2_ Assay, AEGM medium (200 µL) and H_2_O_2_ substrate solution (50 µL) were added on the surface of the cells. After 3 h incubation at 37 °C/5% CO_2_, 75 µL of the solution was transferred into a white 96 well plate. Detection solution (75 µL) was added and after 20 min incubation at room temperature, the relative luminescence was measured. 

In order to measure the cell viability in the same cell culture insert, the remaining medium was removed from the cells, and 300 µL AEGM medium as well as 60 µL CellTiter-Blue^®^ solution were added. The cultures were then incubated for 2 h at 37 °C/5% CO_2_. Afterwards, 100 mL of the solution were pipetted into a black 96 well plate to measure the fluorescence at 544_Ex_/590_Em_ nm. 

### 2.5. Statistical Analysis

Significant differences between two groups were evaluated by Student’s unpaired *t*-test, whereas the symbols (asterisks or hash marks) are defined as followed: ****, ^####^ *p* < 0.0001; ***, ^###^ *p* = 0.0001–0.001; **, ^##^ *p* = 0.001–0.01; *, ^#^ *p* = 0.01–0.05. 

## 3. Results

**Figure 1:**
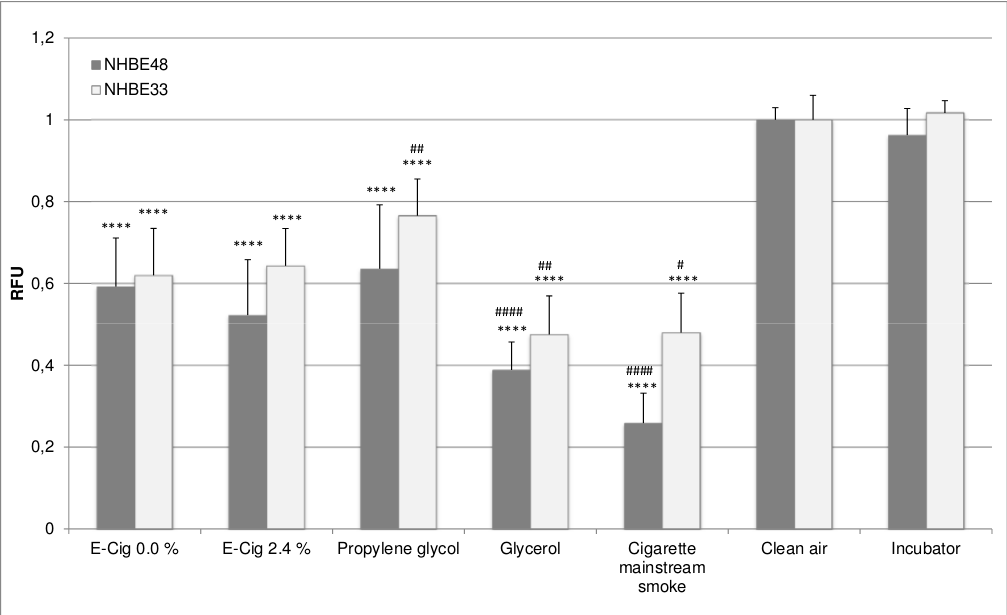
Cell viability of NHBE cells after exposure. The results are given as mean of five (NHBE48) and three (NHBE33) independent experiments with three samples each + standard deviation. The asterisks indicate the statistical significance compared to clean air exposed cells, the hash marks compared to cells exposed to e-cigarette vapor with 0% nicotine. The relevance of the significance is explained in Section 2.5.

**Figure 2:**
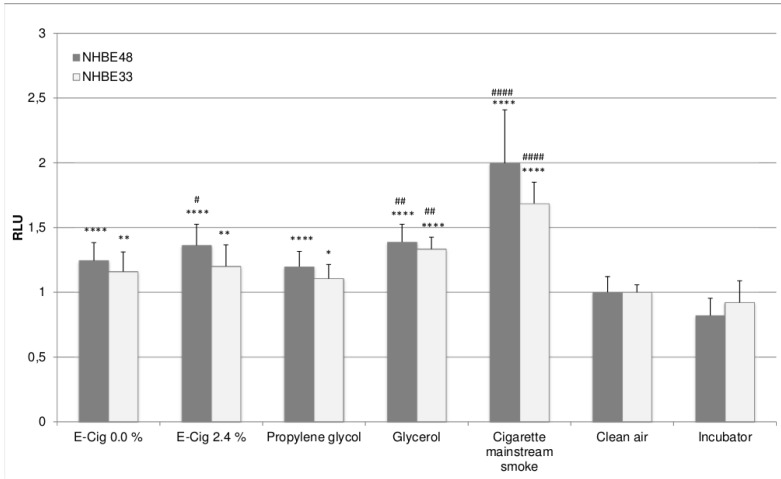
Oxidative stress levels in NHBE cells after exposure. The results are given as mean of five (NHBE48) and three (NHBE33) independent experiments with three samples each + standard deviation. The asterisks indicate the statistical significance compared to clean air exposed cells, the hash marks compared to cells exposed to e-cigarette vapor with 0% nicotine. The relevance of the significance is explained in Section 2.5.

Figure 1 shows the cell viability of the exposed NHBE cells, Figure 2 shows the corresponding oxidative stress levels. The results were obtained by performing five independent exposure experiments in case of NHBE48 cells and three independent experiments in case of NHBE33 cells with three samples each for every experimental group. For comparability, all results were normalized to the values of the clean air control. 

Compared to clean air exposed cells, cells exposed to e-cigarette vapor show significantly lower cell viability as well as higher oxidative stress levels. In case of NHBE33 cells, these results are comparable for both tested liquids, independent of the nicotine concentration. In NHBE48 cells, the exposure to nicotine containing e-liquid vapor led to higher oxidative stress, whereas no difference was seen in the cell viability. 

Cells exposed to 200 puffs of pure propylene glycol also show reduced cell viability and higher oxidative stress levels than clean air exposed cells. The levels are comparable to e-cigarette vapor exposed cells, only for NHBE33 cells, the viability of propylene glycol exposed cells is higher than that of e-cigarette vapor exposed cells. Cells exposed to glycerol show a significantly reduced viability compared to clean air as well as e-cigarette vapor exposed cells. 

Cigarette mainstream smoke treated cells shows the highest oxidative stress levels in NHBE cells from both donors as well as the lowest cell viability in NHBE48 cells. In NHBE33 cells, the cell viability is comparable to that of glycerol exposed cells. However, it needs to be considered, that the number of puffs taken was not identical for K3R4F cigarettes and glycerol. In order to compare the effects of e-cigarette vapor liquid exposure and mainstream smoke exposure, the dosages have to be considered. During the e-cigarette exposure, the cells were exposed to vapor from 200 puffs. The mainstream smoke exposure was done with 10 cigarettes, each puffed six times. The puff volume and the dilution were identical in both cases, and were therefore not factored in the calculations. 

**Figure 3:**
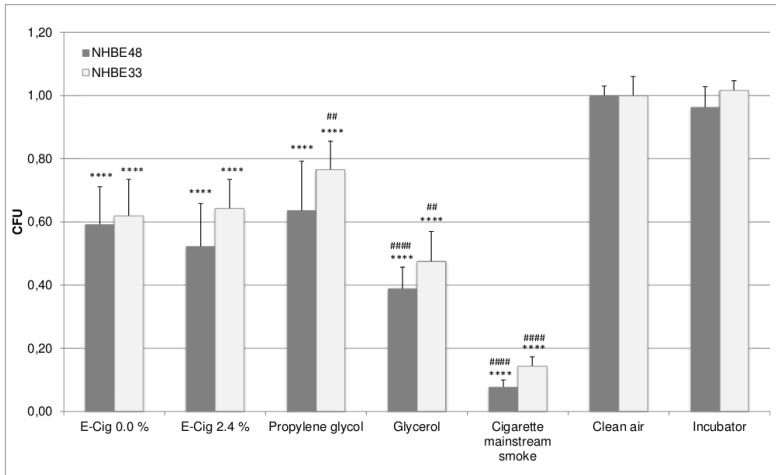
Puff-adjusted values for cell viability of NHBE cells after exposure. The results are given as mean of five (NHBE48) and three (NHBE33) independent experiments with three samples each + standard deviation. The asterisks indicate the statistical significance compared to clean air exposed cells, the hash marks compared to cells exposed to e-cigarette vapor with 0% nicotine. The relevance of the significance is explained in Section 2.5.

**Figure 4:**
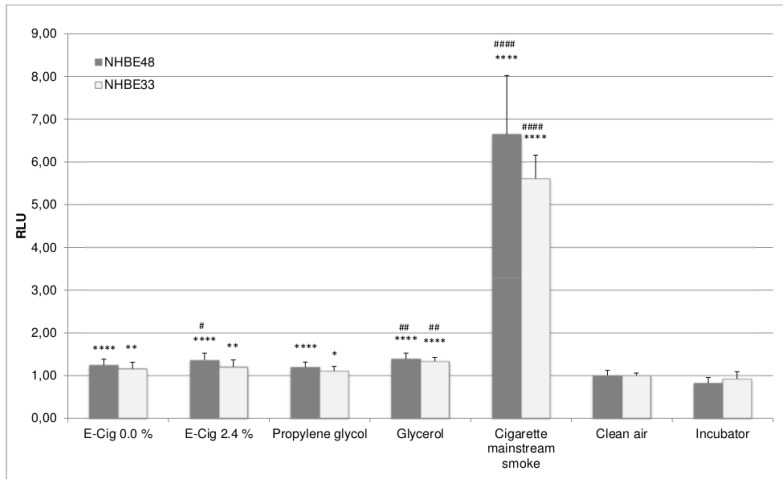
Puff-adjusted values for oxidative stress levels in NHBE cells after exposure. The results are given as mean of five (NHBE48) and three (NHBE33) independent experiments with three samples each + standard deviation. The asterisks indicate the statistical significance compared to clean air exposed cells, the hash marks compared to cells exposed to e-cigarette vapor with 0% nicotine. The relevance of the significance is explained in Section 2.5.

The ratio between e-cigarette and cigarette puffs was 200/60 = 3.33. Since the cell viability decreases with increasing amount of smoked cigarettes, the results were divided by 3.33. The oxidative stress level increases with the amount of cigarettes; therefore the results were multiplied by 3.33. The puff-adjusted values are shown in Figure 3 and Figure 4. After adjustment, the cell viability differs by a factor of 4.5–8 and the oxidative stress levels by factor 4.5–5 between mainstream smoke and e-cigarette vapor exposed cells, depending on the cell donor. 

Due to the fact that all exposure experiments other than the mainstream smoke exposure was performed using the same puff numbers, no adjustment was conducted here. Therefore the differences between the results of e-cigarette liquid vapor, propylene glycol and glycerol exposure were the same as before. 

In general, the viability of NHBE33 cells is slightly higher and oxidative stress levels are slightly lower than those of NHBE48 cells. However, this can be found in all experimental groups and can therefore be attributed to donor specific variations. Despite those slightly different absolute values, the overall result is the same in NHBE cells of both donors, meaning that the effects in the experimental groups show the same results relative to each other. 

## 4. Discussion and Conclusions

Our results clearly demonstrate that the tested e-cigarette vapor had toxicological effects on primary NHBE cells when exposed directly at the air-liquid interface. Interestingly, the presence of nicotine had no effect on the cell viability and only the cells of one donor showed higher oxidative stress levels after the exposure to vapor of an e-cigarette liquid containing 2.4% nicotine compared to nicotine-free liquid. In both NHBE cells, the cell viability decreased significantly and the oxidative stress levels increased significantly in e-liquid vapor exposed cells compared to clean air exposed cells. However, compared to mainstream smoke exposed cells, the values were significantly higher (cell viability) respectively lower (oxidative stress). After adjusting the results to the number of puffs taken during the exposure, the differences were even more pronounced; the cell viability was 4.5–8 times higher and the oxidative stress levels 4.5–5 times lower, depending on the cell donor. We could also show, that the pure carrier substances propylene glycol and glycerol exhibited toxicological effects. However, in case of using the carrier substances as reference standard against a tested e-cigarette liquid, the concentrations of the carriers should be adjusted to the concentration in the liquid. 

Our results are in accordance with the *in vitro* studies of Farsalinos *et al.* and Romagna *et al.*, using extracts of e-cigarette vapor and mainstream smoke on myocardioblasts and fibroblasts [12,13]. However, myocardioblasts and fibroblasts, certainly also affected by those substances, are normally not in direct contact with the native vapor like bronchial epithelial cells; metabolic products are of greater importance there. Our data suggests, that bronchial epithelial cells as main target cells are a sensitive model to evaluate toxicological effects of e-cigarette vapor in an *in vivo*-like environment. These findings are an important aspect for hazard identification. 

In our study, we used undifferentiated bronchial epithelial cells, consisting of a mixed cell population including a large proportion of basal cells but also progenitor cells for secretory and ciliated cells. When cultivated under air-lift conditions, these cultures are able to fully differentiate into cultures with *in vivo*-like phenotype with ciliated cells, club cells, and goblet cells among others. We chose the early stage of the cells for our exposure experiments to show the effects of e-cigarette vapor on a regenerating epithelium, like found after injury. Further studies will comprise experiments of fully differentiated cells with single and repeated exposure. 

However, comparing the results from e-cigarette vapor to mainstream smoke exposed cells, the interpretation of the outcome is still challenging. Combustible cigarettes are smoked in exposure experiments according to ISO 3308, meaning a puff volume of 35 mL, a puff time of 2 s, and an interpuff interval of 60 s, but these smoke parameter do not resemble the vaping topography in e-cigarette users. Farsalinos *et al.* studied the smoke behavior of 45 experienced e-cigarette users and 35 smokers with regard to puff, inhalation and exhalation duration. They found higher puff duration, lower inhalation time and no difference in exhalation duration. Based on the results, suggestions for exposure parameter are four-second puffs with an interval of 20–30 s [14]. In our study, we used 2 s puffs, which were puffed successively. Further studies will be made to evaluate the effects of e-cigarette vapor when exposed using the suggested parameters. 

The reference to puff numbers is a first approach to compare the toxicity of combustible cigarettes and e-cigarettes. However, the number of puffs taken by users seem to differ extremely between e-cigarettes and combustible cigarettes. A survey on www.vaping.com in 2014 showed, that, for example, 22.6% of e-cigarette users consume 4–5 mL e-liquid per day and 9.9% even more than 8 mL [15]. The e-cigarette we used in our studies can be filled with 1.5 mL, which is adequate for about 400 puffs. A daily consumption of 4.5 mL would be enough for about 1200 puffs. A heavy smoker, who consumes combustible cigarettes might do about 300 puffs, if smoking 30 cigarettes with 10 puffs each, giving a factor of four between daily puffs done with e-cigarettes or combustible cigarettes. Furthermore, it needs to be considered, that various e-cigarette types and generations produce different volumes of vapor and that atomizers with different strength may produce other byproducts. 

However, if the toxicity of e-cigarette vapor and cigarette mainstream smoke is compared on basis of the same smoking parameters as well as the same number of puffs, the cell viability is about 4.5–5 times lower and the oxidative stress levels 4.5–5 times higher in combustible cigarettes. Our results show, that the used experimental setting is suitable to obtain reproducible, reliable toxicological data about e-cigarette vapor. The *in vitro* testing of e-cigarette vapor provides the opportunity to test e-cigarette liquids for their toxicity to ensure the safety of the users. The *in vitro* analysis using human bronchial epithelial cells is an important testing method, since it can give reliable data about the toxicological effects in the human body and should therefore be a standard in e-cigarette liquid production and quality control. 
